# The impact of occupational hazards in coking plants on the incidence of hypertension—a longitudinal study

**DOI:** 10.3389/fpubh.2026.1762496

**Published:** 2026-02-03

**Authors:** Wei Zhang, Shengyu Fan, Yifan Li, Tiantian Chen, Xingyu Peng, Hongmei Gu, Shu Guo

**Affiliations:** 1 State Key Laboratory of Environmental Pollution Health Risk Assessment, South China Institute of Environmental Sciences, Ministry of Ecology and Environment, Guangzhou, China; 2 School of Public Health, Mudanjiang Medical University, Mudanjiang, China; 3 School of Public Health, China Medical University, Shenyang, China; 4 Loudi Center for Disease Control and Prevention, Loudi, China

**Keywords:** benzene and benzene series, coking plant worker, dust, hypertension, noise, occupational hazards

## Abstract

**Objective:**

To explore the incidence density and risk of hypertension among employees in different workshops of a coking plant.

**Method:**

The research subjects were employees of a coking plant in western Inner Mongolia. Based on inclusion and exclusion criteria, we studied 448 employees hired between 2011 and 2023. Of these, 285 were front-line workers exposed to occupational hazards, while 163 were second-line workers with lesser or no exposure. We collected data on the general demographic characteristics, systolic blood pressure, diastolic blood pressure, and other information of the research subjects, calculated the incidence density of hypertension, and used the Cox proportional hazards regression model to analyze the association between exposure to occupational risk factors and the risk of hypertension.

**Results:**

During the observation period, we identified 229 cases of hypertension, with an incidence density of 11,187.10 per 100,000 person-years. The average age of the patients was 42.33 ± 8.24 years, and the average working duration was 3.57 ± 2.84 years. The number of new cases in logistics, coal preparation, chemical production, and coking workshops were 53, 45, 47, and 84, respectively, with incidence densities of 7,019.83, 14,469.45, 12,737.13, and 13,725.49 per 100,000 person-years, respectively. The risk of hypertension in coal preparation, chemical production, and coking workshops was 4.061, 3.364, and 2.427 times higher than that in logistics workers, respectively. Cox regression analysis showed that, after controlling for gender, age, smoking, drinking, and other factors, the risk ranking of hypertension in each workshop was as follows: coking workshop (1.822) > chemical production workshop (1.752) > coal preparation workshop (1.622). The log-rank test revealed that the differences in disease-free survival distribution among workers in different workshops were statistically significant (*p* < 0.05).

**Conclusion:**

The study shows that the incidence density of hypertension among employees in the chemical industry, coking, and coal preparation workshops after joining the company is significantly higher than that in the logistics group, and the risk of incidence is relatively higher (HR = 1.622–1.822 after controlling for confounding factors). Long-term exposure of coking plant workers to related environments increases the risk of hypertension.

## 1 Introduction

Hypertension, one of the chronic diseases with the highest incidence globally, has become a major public health challenge. In 2024, there were 1.4 billion people with hypertension worldwide. The prevalence of hypertension among Chinese adults exceeds 27%, with an increasing trend among younger populations. The cardiovascular complications it causes—such as stroke and coronary heart disease—seriously threaten public health and labor productivity (1). The occupational environment is a key setting for long-term adult exposure, and the relationship between occupational hazards and hypertension has become a central focus in occupational epidemiology research. Among these, the potential cardiovascular damage resulting from combined occupational exposures in industrial settings is particularly pronounced.

China is the world’s largest coke producer. In 2023, its output reached 470 million tons, accounting for 70% of the global total and representing a year-on-year increase of 3.6%. The coking industry plays a critical role in foundational sectors such as steel and chemicals. However, the coking process is accompanied by the emission of a complex and diverse range of occupational hazards, creating an environment characterized by multi-pathway and multi-type exposures. Chemical pollutants include not only traditional particulate matter (TSP, PM2.5, coal dust), sulfur dioxide, and nitrogen oxides, but also volatile organic compounds (VOCs) such as benzene, toluene, xylene, naphthalene, phenols, hydrogen sulfide, and ammonia. Additionally, strongly carcinogenic polycyclic aromatic hydrocarbons such as benzo(a)pyrene are present. Some pollutants—such as lead, cadmium, arsenic, and fluorine—can also contribute to composite pollution by adsorbing onto particulate matter (2, 3). Physical hazards such as workplace noise, high temperatures, and prolonged exposure to vibration further exacerbate health risks (4). These hazardous agents enter the human body via multiple pathways—including inhalation, dermal contact, and ingestion—leading to sustained damage across multiple organ systems.

In recent years, occupational epidemiological studies have confirmed that the health effects of occupational hazards in coking plants have extended beyond traditional respiratory diseases (such as pneumoconiosis and chronic bronchitis) and skin damage, now encompassing the cardiovascular system and other organ systems (5). Building on these findings, this study selected 448 employees who were employed at a coking plant in western Inner Mongolia between 2011 and 2023 as research subjects. Over a 12-year longitudinal follow-up period, we systematically analyzed occupational hazard exposure levels in different operational areas—including coal preparation, coking, and chemical production. We explored the incidence patterns and risk differences of hypertension among employees in each workshop, and identified key factors linking occupational exposure to hypertension onset. The findings aim to elucidate the mechanisms by which combined occupational hazards in coking plants damage the cardiovascular system. They also provide a scientific basis for developing targeted occupational health protection strategies and establishing early monitoring systems for high-risk groups. Ultimately, this research holds practical significance for reducing the incidence of occupational hypertension, safeguarding workers’ health rights, and promoting sustainable enterprise development.

## 2 Research objects and methods

### 2.1 Research objects

All employees of a coking plant in western Inner Mongolia Autonomous Region were considered as potential study subjects. The inclusion criteria were as follows: (1) aged ≥ 20 years; (2) employed at the coking plant for more than 1 year; (3) no personal or family history of cardiovascular disease prior to employment; (4) complete health examination records from 2011 to 2023. The exclusion criteria were as follows: (1) diagnosis of hypertension (including grade 1, 2, or 3) prior to employment, or documented history of hypertension in medical records; (2) presence of organic diseases known to affect blood pressure, including renal disease (e.g., chronic nephritis, renal insufficiency), endocrine disorders (e.g., hyperthyroidism, diabetes), cardiovascular disease (e.g., coronary heart disease, heart failure, congenital heart disease), or neurological conditions (e.g., stroke, intracranial tumor); (3) incomplete personal health examination data. A total of 489 employees were hired at the plant between 2011 and 2023. After excluding 15 individuals with abnormal blood pressure at baseline, 10 with a family history of hypertension, and 16 with less than 1 year of employment, 448 employees were ultimately enrolled as study subjects. Of these, 285 were frontline workers with direct occupational exposure (76 in chemical production, 141 in coking, and 68 in coal preparation), and 163 were administrative or support staff with minimal or no occupational exposure (including quality planning, safety and environment, dispatch, mechanics, and electricians).

### 2.2 Research methods

We collected longitudinal health examination records of 489 employed workers from 2011 to 2023, including pre-employment and annual follow-up examinations. Data were extracted from 8 March to 1 May 2024. All records were double entered using EpiData 3.1. Variables included age, sex, years of service, smoking (≥6 consecutive months), alcohol consumption (≥3 times per week), family history of cardiovascular disease, and blood pressure. Researchers had no access to personally identifiable information at any stage. Using routine workplace hazard reports provided by the coking enterprise, we collected air-monitoring data for dust, noise, carbon monoxide, hydrogen sulfide, benzene and benzene homologs, phenol, naphthalene, ammonia, and nitrogen oxides. Mobile monitoring campaigns were conducted in March and October 2023, March and October 2024, and July 2025. Qualitative and quantitative analyzes of benzene and its homologs were performed by gas chromatography–mass spectrometry. The study protocol was approved by the Medical Ethics Committee of the South China Institute of Environmental Sciences, Ministry of Ecology and Environment (Approval No. South China Institute of Medical Ethics [2025] 9). Written informed consent was obtained from every participant. The observation period began on 1 January 2011 and ended on 31 December 2023. The primary outcome was incident hypertension identified during the annual occupational health examination.

Hypertension was defined according to the China Guidelines for the Prevention and Treatment of Hypertension (2024 revision). Participants remained free of antihypertensive medication throughout follow-up. On three separate visits, seated blood pressure was measured with an automated oscillometric device (Omron HEM-7300) after 5 min of rest; smoking, alcohol, caffeine, strenuous exercise and emotional excitement were avoided for ≥30 min beforehand. The arm was supported at heart level, and three readings were taken 1 min apart; the mean of the last two was recorded. Hypertension was diagnosed when mean systolic blood pressure was ≥140 mmHg and/or diastolic blood pressure ≥90 mmHg. Annual examination records verified that none of the 448 participants used antihypertensive drugs at any time during the observation period, satisfying the drug-free criterion for diagnosis.

### 2.3 Statistical analysis

EpiData 3.1 software was used to build the database, and all the occupational health records monitoring data of employees who entered the factory from 2011 to 2023 were double entered to avoid possible errors during the data entry process. SPSS 26.0 software was used to carry out statistical analysis. Measurement data were expressed as mean ± standard deviation ( $\bar{x}$ ± s). Comparisons between three groups or more were performed using one-way ANOVA tests. Count data were described using rates or constituent ratios, and the χ^2^ test or Fisher’s exact probability method was used to compare rates. Cox proportional regression model was used to analyze the association between exposure to occupational harmful factors and the incidence of hypertension in coking plant workers, and the HR (95% CI) value of hypertension incidence was calculated. Log-rank test was used to analyze the differences in survival distributions among the four groups. *p* < 0.05 (two-sided test) indicates a statistical difference.

## 3 Results

### 3.1 Basic situation

Age, sex, smoking status, alcohol consumption, systolic blood pressure (SBP) and diastolic blood pressure (DBP) were taken as study variables; baseline characteristics are shown in Table 1. Mean age and SBP were highest in coal-preparation workers, followed by those in coking, chemical production and logistics. DBP decreased in the order coking > coal preparation > chemical production > logistics. The proportion of males decreased from coking to coal preparation, chemical production and logistics. Smoking prevalence was coking > coal preparation > logistics > chemical production (*p* < 0.05, χ^2^ test).

**Table 1:** Pre-employment characteristics of logistics, coal preparation, chemical production, and coking employees [n (%) or $\bar{x}$ ± s].

Characteristic	Logistics	Coal preparation	Chemical production	Coking	F or χ^2^	*p*
	n% or $\bar{x}$ ± s	n% or $\bar{x}$ ± s	n% or $\bar{x}$ ± s	n% or $\bar{x}$ ± s		
Age/years	33.24 ± 8.017	42.34 ± 7.732	36.79 ± 9.235	40.49 ± 7.234	30.763	<0.001
≤40	127(77.91)	21(30.88)	48(63.16)	65(46.10)	56.176	<0.001
>40	36(22.09)	47(69.12)	28(36.84)	76(53.90)		
Gender						
Male	106(65.03)	49(72.06)	51(67.11)	119(84.40)	15.703	0.001
Female	57(34.97)	19(27.04)	25(32.89)	22(15.60)		
Smoking						
Yes	63(38.65)	33(48.5)	27(35.53)	76(53.90)	10.231	0.017
No	100(61.35)	35(51.47)	49(64.47)	65(46.10)		
Alcohol consumption						
Yes	30(18.40)	17(25.00)	11(14.47)	32(22.70)	3.399	0.334
No	133(81.60)	51(75.00)	65(85.53)	109(77.30)		

### 3.2 Exposure levels to occupational hazards

We collected routine monitoring data on occupational hazard factors for workers in coal preparation, chemical production, and coking workshops. Logistics employees were not tested because they had little or no exposure to occupational hazards, so there is no relevant data. The results showed that in the workplaces of workers in the coal preparation workshop, 84.94 and 27.23% of coal dust and other dust were, respectively, higher than the occupational exposure limit specified in the “Exposure Limits to Hazardous Factors in the Workplace Part 1: Chemical Hazardous Factors” (GBZ2.1–2019; coal dust 4 mg/m^3^, other dust 8 mg/m^3^). In the workplaces of employees in the coking workshop, 87.10 and 28.89% of coal dust and other dusts were, respectively, higher than the occupational exposure limit. Mobile monitoring found that the concentration of benzene and naphthalene in the coke oven area and chemical production area exceeded the occupational exposure limits (6 mg/m^3^, 50 mg/m^3^). The noise in the workplaces of workers in the coal preparation, chemical industry, and coking workshops was 11.96, 16.94, and 10.32%, respectively, higher than the occupational exposure limit (85 dB) specified in the “Exposure Limits to Hazardous Factors in the Workplace Part 2: Physical Hazardous Factors” (GBZ2.2–2007). See Tables 2, 3 for details.

**Table 2:** Exposure levels of occupational hazardous factors in various workshops of the coking plant from 2011 to 2023.

Detection factor	Occupational exposure limit (PC-TWA)	Coal preparation			Chemical production			Coking		
		Test results	Detection points	Excessive points	Test results	Detection points	Excessive points	Test results	Detection points	Excessive points
Coal dust/(mg/m^3^)	4	0.50–18.30	631	536	/	/	/	0.50–14.10	93	81
Other dust/(mg/m^3^)	8	0.30–12.70	235	64	/	/	/	0.50–12.00	45	13
Noise/dB	85	70.50–88.60	443	53	65.30–92.40	419	71	62.10–89.85	436	45
Carbon monoxide/(mg/m^3^)	20	/	/	/	0.50–14.20	135	0	0.70–18.20	384	0
Hydrogen sulfide/(mg/m^3^)	10	/	/	/	0.60–12.30	150	0	0.60–15.90	137	0
Benzene/(mg/m^3^)	3	/	/	/	<0.60	132	0	<0.60	50	0
Toluene/(mg/m^3^)	50	/	/	/	<1.00	132	0	<1.00	50	0
Xylene/(mg/m^3^)	50	/	/	/	<3.00	132	0	<3.00	50	0
Cresol/(mg/m^3^)	2	/	/	/	0–0.35	132	0	<0.22	50	0
Phenol/(mg/m^3^)	2	/	/	/	0–0.94	132	0	<0.22	50	0
Naphthalene/(mg/m^3^)	50	/	/	/	<0.30	115	0	<0.30	30	0
NOx/(mg/m^3^)	5	/	/	/	0.19–1.19	144	0	0.18–1.23	68	0
Ammonia/(mg/m^3^)	20	/	/	/	0.76–15.1	115	0	/	46	0

/, indicates that occupational disease hazard factors are not detected in routine monitoring.

**Table 3:** Qualitative and quantitative analysis of benzene and benzene series in coal preparation, chemical production, and coking areas from 2023 to 2025.

Detection factor	Occupational exposure limit (PC-TWA)	Coal preparation area	Coke oven area	Chemical production area
Benzene /(mg/m^3^)	6	3.66–4.52	32.53–55.30	48.84–117.40
Toluene/(mg/m^3^)	50	4.51–6.52	4.69–33.56	2.51–76.60
Xylene/(mg/m^3^)	50	3.68–4.21	3.56–4.88	2.99–3.58
Naphthalene /(mg/m^3^)	50	1.65–2.88	15.85–51.20	35.20–76.40
1-Methylnaphthalene/(mg/m^3^)	/	2.32–4.21	4.65–7.99	6.80–9.17

### 3.3 The incidence of hypertension among employees in coking plants

Between 2011 and 2023, 229 incident cases of hypertension were identified, yielding an incidence density of 11,187.10 per 100,000 person-years. The mean age at diagnosis was 42.33 ± 8.24 years, and the mean duration of employment was 3.57 ± 2.84 years. The numbers of new-onset cases in logistics, coal preparation, chemical production, and coking were 53, 45, 47, and 84, respectively, with corresponding incidence densities of 7,019.83, 14,469.45, 12,737.13, and 13,725.49 per 100,000 person-years. The risk of developing hypertension among workers in coal preparation, chemical production, and coking was 4.061-fold, 3.364-fold, and 2.427-fold higher, respectively, than that among logistics workers. Men had 3.292-fold higher risk than women, participants aged >40 years had 2.634-fold higher risk than those aged ≤40 years, current smokers had 1.761-fold higher risk than never smokers, and current drinkers had 1.768-fold higher risk than non-drinkers. These findings indicate that male sex, age >40 years, smoking, and alcohol consumption are risk factors for incident hypertension (Table 4).

**Table 4:** Shows the incidence of hypertension among employees in coking plants from 2011 to 2023.

Characteristic	Number of new cases	Person-years of observation	Incidence density/ (per 100,000 person-year)	χ^2^	*p*	RR (95% CI)
Gender						
Female	37	603	6135.99			1.000
Male	192	1,444	13296.40	28.995	<0.001	3.292 (2.110, 5.135)
Age/years						
≤40	74	849	8716.14			1.000
>40	155	1,198	12938.23	24.893	<0.001	2.634 (1.794, 3.869)
Smoking						
No	123	1,094	11243.14			1.000
Yes	106	953	11122.77	8.793	0.003	1.761 (1.210, 2.562)
Drinking						
No	167	1,652	10108.96			1.000
Yes	62	395	15696.20	6.103	0.013	1.768 (1.122, 2.788)
Workshop						
Logistics	53	755	7019.87			1.000
Coal Preparation	45	311	14469.45	22.258	<0.001	4.061 (2.229, 7.398)^*^
Chemical Production	47	369	12737.13	18.319	<0.001	3.364 (1.908,5.931)^*^
Coking	84	612	13725.49	14.154	<0.001	2.427(1.523,3.867)^*^

*Compared with logistics.

### 3.4 The changes in blood pressure levels of employees in coking plants

Changes in blood pressure from before to after hire are detailed in Table 5. Systolic and diastolic blood pressures (SBP/DBP) increased in logistics, coal preparation, chemical production and coking workers. At the end of follow-up, the mean increases in both SBP and DBP among employees in coal preparation, chemical production and coking were greater than those in logistics workers (*p* < 0.05).

**Table 5:** Changes in blood pressure levels of employees in coking plants ( $\bar{x}$ ± s, mmHg).

Time points	Logistics	Coal preparation	Chemical production	Coking	F	*p*
	$\bar{x}$ ± s	$\bar{x}$ ± s	$\bar{x}$ ± s	$\bar{x}$ ± s		
Pre-employment						
Systolic blood pressure	119.74 ± 11.187	124.29 ± 10.239	120.38 ± 11.424	122.79 ± 10.486	3.852	0.010
Diastolic blood pressure	75.74 ± 8.101	78.94 ± 7.707	76.80 ± 7.898	79.04 ± 7.179	5.692	*p* < 0.001
End of observation						
Systolic blood pressure	125.00 ± 15.708	134.78 ± 15.724	130.67 ± 15.223	133.70 ± 17.490	9.600	*p* < 0.001
Diastolic blood pressure	78.99 ± 11.783	86.01 ± 12.776	81.05 ± 12.172	86.36 ± 11.564	11.908	*p* < 0.001
Pre-entry—end of observation						
Systolic blood pressure	5.26 ± 14.67	10.49 ± 16.34	10.29 ± 14.83	10.90 ± 16.96	4.085	0.007
Diastolic blood pressure	3.25 ± 11.47	7.07 ± 12.24	4.58 ± 12.52	7.14 ± 11.39	3.420	0.017

### 3.5 Analysis of survival data of hypertension incidence among employees in coking plants

In order to further explore the association between occupational hazard exposure and incident hypertension among coking plant employees, we conducted multivariable analysis using the Cox proportional hazards regression model. After testing the proportional hazards assumption (*p* > 0.05), all covariates—dust and other occupational exposures, sex, age, smoking, and alcohol consumption—met the assumption of equal proportional hazards.

We used exposure to occupational hazards (dust as the main surrogate) as the independent variable and adjusted for sex, age, smoking, and alcohol consumption as covariates. Table 6 summarizes the findings. Without adjustment, workers in coal preparation, chemical production, and coking had hazard ratios (HRs) for hypertension of 1.943, 1.810, and 1.915, respectively, compared with logistics workers. After adjustment for sex and age, the corresponding HRs were 1.656, 1.742, and 1.622. With full adjustment (sex, age, smoking, and drinking), the ranking remained coking (HR = 1.822) > chemical production (HR = 1.752) > coal preparation (HR = 1.622).

**Table 6:** Cox regression analysis of the risk of hypertension among employees in coking plants.

Occupational exposure	Workshop	B	SE	Wald χ^2^ value	HR (95% CI)	*p*
Model 1^a^	Coal Preparation	0.664	0.203	10.682	1.943 (1.305, 2.895)	0.001
	Chemical Production	0.593	0.201	8.747	1.810 (1.221, 2.681)	0.003
	Coking	0.605	0.176	13.644	1.915 (1.357, 2.681)	<0.001
	Coal Preparation	0.505	0.226	4.983	1.656 (1.063, 2.579)	0.026
Model 2^b^	Chemical Production	0.555	0.205	7.359	1.742 (1.166, 2.601)	0.007
	Coking	0.571	0.197	8.422	1.771 (1.204, 2.604)	0.004
Model 3^c^	Coal Preparation	0.484	0.230	4.419	1.622 (1.033, 2.547)	0.036
	Chemical Production	0.561	0.204	7.542	1.752 (1.174, 2.615)	0.006
	Coking	0.600	0.199	9.114	1.822 (1.234, 2.690)	0.003

a Covariates are not controlled.

b Gender and age are controlled.

c Gender, age, smoking and drinking are controlled.

**Figure 1:**
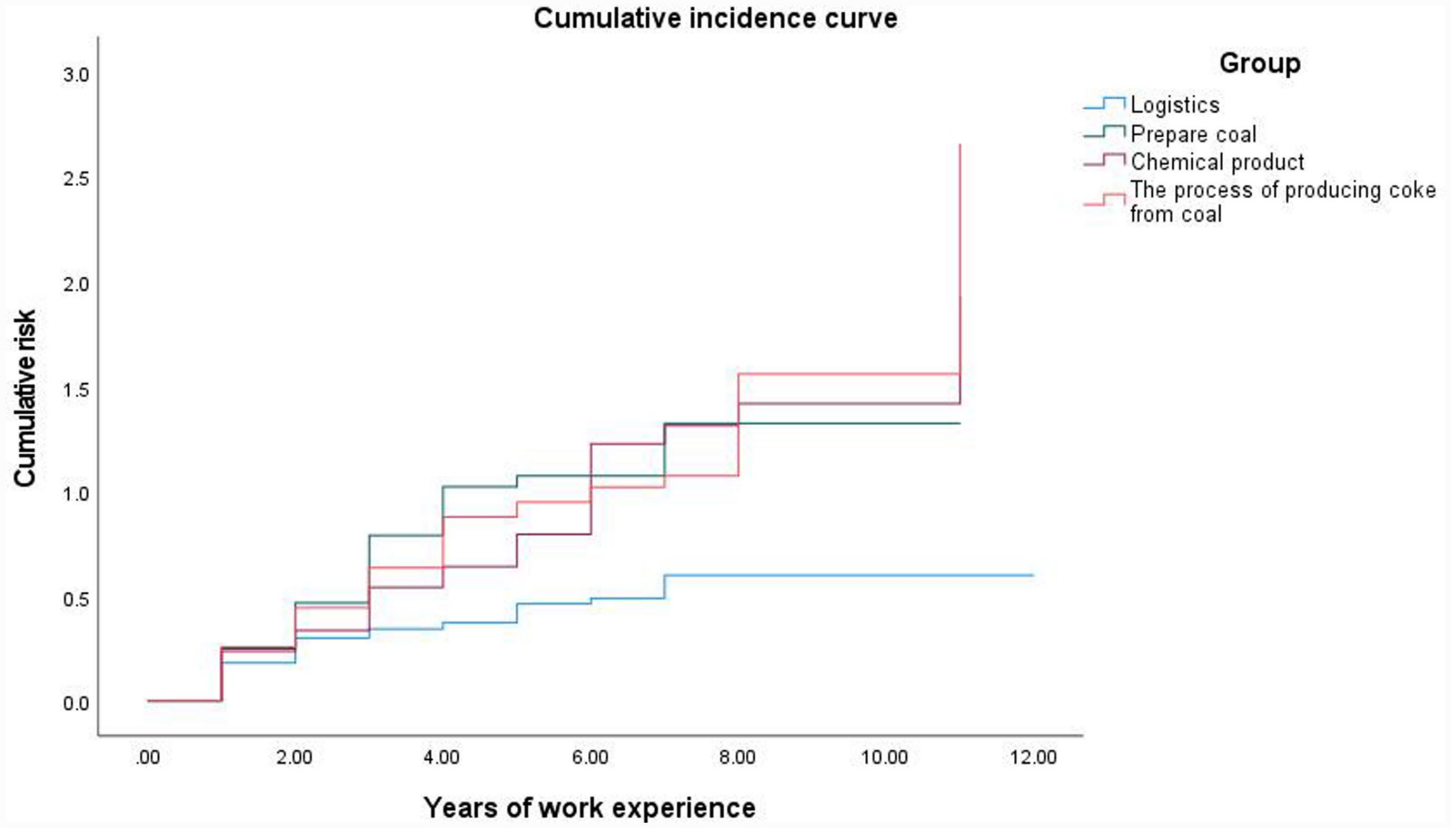
The cumulative incidence curve of hypertension among employees in coking plants.

The median time-to-hypertension could not be estimated for logistics workers because fewer than 50% experienced the event during follow-up. Among coal-preparation, chemical-production and coking workers, the median (95% CI) hypertension-free survival was 3.000 (2.092–3.908), 5.000 (3.696–6.304) and 4.000 (3.195–4.805) years, respectively (Table 7). Log-rank tests showed that hypertension-free survival differed significantly across groups (*p* < 0.05). Figure 1 shows that cumulative incidence increased most rapidly in coking workers, followed by coal-preparation and chemical-production workers, and remained lowest in logistics employees. Over time, the between-group gap widened, suggesting that occupational exposure accelerates hypertension onset. The early and persistent separation of survival curves underscores the importance of early intervention in high-risk occupational groups.

**Table 7:** Analysis of survival time without hypertension in different groups.

Group	$\bar{x}$ (95%CI)	M (95%CI)
Logistics	7.963 (7.079, 8.828)	/
Coal preparation	4.970 (3.969, 5.971)	3.000 (2.092, 3.908)
Chemical production	5.356 (4.411, 6.302)	5.000 (3.696, 6.304)
Coking	5.083 (4.357, 5.809)	4.000 (3.195, 4.805)

## 4 Discussion

### 4.1 Analysis of workplace differences in hypertension caused by occupational exposure

The research results showed that the incidence density of hypertension in front-line workshops (12,737.13-14,469.45 per 100,000 person-years) is significantly higher than that in back-end workshops (7,019.83 per 100,000 person-years), and the coking workshop has the highest risk (HR = 1.822) after controlling for confounding factors. This is closely related to the pollutant exposure characteristics and combined injury mechanisms of each workshop.

The rates of excessive coal dust in coal preparation and coking workshops are 84.94 and 87.10%, respectively. After these particles are inhaled through the respiratory tract, they can activate alveolar macrophages to release pro-inflammatory factors such as IL-6 and TNF-*α*, and at the same time generate reactive oxygen species (ROS) through the Fenton reaction, causing oxidative stress damage (3, 6). Hai et al.’s study confirmed that coal dust exposure could significantly increase the levels of C-reactive protein and 8-isoprostane F2α in the peripheral blood of workers, leading to decreased vascular elasticity through the destruction of vascular endothelial cells (VEC) and inhibition of endothelial repair ability (7). This explains why, although there is no obvious excess of benzene series substances in the coal preparation workshop, there is still a high risk of disease due to high dust exposure. Mobile monitoring showed that the benzene concentration (48.84–117.40 mg/m^3^) and naphthalene concentration (35.20–76.40 mg/m^3^) in the chemical production area far exceeded the occupational exposure limit, and similar pollution was also found in the coking workshop. Zhou Bingxian et al. confirmed that exposure to low-concentration mixed benzene could reduce the production of nitric oxide (NO) by inhibiting the activity of endothelial nitric oxide synthase (eNOS). NO is a key vasodilator factor and its insufficient synthesis can lead to increased vascular smooth muscle contraction and directly increase blood pressure (8). The fat-soluble properties of naphthalene can enhance tissue penetration, aggravate VEC apoptosis, and synergize with benzene compounds to amplify the damage effect (9). This was also the core reason why the risk of the chemical production workshop (HR = 1.752) was second only to the coking workshop.

There were high concentrations of coal dust, benzene compounds, and naphthalene exposure simultaneously in the coking workshop, which formed a synergistic effect of “inflammatory damage and vasodilation inhibition,” and was superimposed by physical factors such as noise, further amplifying the risk (10). Liang Jiaojun et al. found that when polycyclic aromatic hydrocarbons (PAHs) and particulate matter in coke oven emissions were jointly exposed, the risk of hypertension increased 2.1 times compared with single exposure, which is consistent with the results of this study that the coking workshop has the highest risk (11). The noise exceedance rate in each workshop ranged from 10.32 to 16.94%. Long-term noise exposure can activate the sympathetic nervous system-adrenal medulla axis, leading to excessive secretion of catecholamines, while also upregulating the activity of the renin-angiotensin-aldosterone system (RAAS), causing peripheral vascular constriction and elevated blood pressure (12, 13). Zhou et al. confirmed in a study of occupational populations in southern China that noise exposure has a dose–response relationship with increased blood pressure, and when it is combined with chemical pollutants, the vascular damage effect is significantly enhanced (13). This also explains why the increase in systolic blood pressure in front-line workshops is significantly higher than that in logistics workshops.

### 4.2 Interaction and mechanism of demographic and lifestyle factors

Research shows that the risk of hypertension in men is 3.292 times that of women. This is because male employees are more concentrated in high-exposure workshops, such as coking and coal preparation workshops. This concentration is due to the high intensity of physical labor and the promotion of vascular smooth muscle proliferation by testosterone levels (14). The hazard ratio (HR) for individuals aged >40 years is 2.634. This is not only related to the natural degeneration of vascular elastic fibers and the decline in vascular endothelial cell (VEC) function but also to long-term occupational exposure, which accelerates telomere shortening and exacerbates vascular aging (15). This forms an “age-exposure” synergistic harmful effect, consistent with the average patient age of 42.33 ± 8.24 years.

The HR for smoking employees is 1.761. Nicotine in tobacco smoke can directly damage VECs and induce an increase in CYP1A1 enzyme activity. This accelerates the metabolic activation of polycyclic aromatic hydrocarbons (PAHs) in coke oven emissions, generating more highly toxic intermediates (such as benzo[a] pyrene diol epoxide). These processes further amplify oxidative stress and inflammatory reactions (5, 16). Zhang et al. confirmed that there is a significant interaction between smoking and PAH exposure, which can increase the risk of cardiovascular disease by 2.3 times (5). This finding is consistent with the results of our study.

The HR for drinkers is 1.768. Alcohol inhibits the release of vasodilator factors (such as prostacyclin) and stimulates sympathetic nerve excitation, leading to an acute increase in blood pressure. Long-term drinking induces continuous activation of the renin-angiotensin-aldosterone system (RAAS), which can lead to sustained increases in blood pressure.

### 4.3 Research significance, limitations and prospects

Through a longitudinal design, this study clarified the differences in the risk of hypertension due to occupational composite exposure in different workshops of the coking plant. It revealed the synergistic damage mechanism of “chemical pollutants (coal dust, benzene series, naphthalene) and physical factors (noise)” and provided precise targets for occupational health protection. Based on the results, we recommend implementing the highest level of protection in the coking workshop, focusing on controlling the coordinated exposure of multiple pollutants; strengthening dust control in the coal preparation workshop; prioritizing the adsorption and purification of benzene series and naphthalene in the chemical production workshop; and conducting key monitoring and intervention for men, senior citizens, smokers, and drinkers.

This study has certain limitations. First, it did not detect individual exposure markers (such as urinary hydroxylated polycyclic aromatic hydrocarbons, serum eNOS activity), making it difficult to quantify the individual exposure–effect relationship. Second, it did not include genetic polymorphisms (such as the eNOS gene rs1799983 site), which may miss individual differences in susceptibility (5, 17). Third, relevant data such as BMI, shift status, physical exercise, eating habits, socioeconomic status, high temperature, and occupational stress among coke plant employees were not collected; therefore, no relevant analysis of these factors was conducted. Previous studies have indicated that factors such as BMI, physical exercise, eating habits, socioeconomic status, and occupational stress may increase the risk of hypertension (13–15, 18–20). Future research should collaborate with coking enterprises in different regions to conduct multi-center studies (21), integrate metabolomics and genomics technologies to analyze the molecular pathways of hypertension caused by occupational exposure, and perform intervention studies to verify the effectiveness of protective measures.

## 5 Conclusion

Through 12 years of longitudinal follow-up, this study systematically explored the onset characteristics and influencing factors of hypertension among 448 employees in a coking plant in western Inner Mongolia and clarified the close relationship between occupational composite exposure and the incidence of hypertension. The results showed that the incidence density of hypertension among employees in coal preparation, chemical production, and coking workshops was significantly higher than that in logistics workshops. After controlling for gender, age, smoking, drinking, and other confounding factors, the risk of hypertension in each front-line workshop remained significantly higher (HR = 1.622–1.822), with the coking workshop having the highest risk.

In summary, the core mechanism of hypertension caused by occupational compound exposure is closely related to the inflammation-oxidative stress response and inhibition of vasodilation caused by chemical pollutants (coal dust, benzene series, and naphthalene) and the sympathetic nerve–renin-angiotensin-aldosterone system activated by physical factors (noise). The synergistic effect of multiple factors further amplifies the vascular damage effect. The results of this study provide a scientific basis for the precise prevention and control of occupational hypertension in the coking industry. They suggest the need to formulate differentiated protection strategies based on the pollution characteristics of coking, chemical production, and coal preparation workshops, while focusing on high-risk groups such as men, seniors, smokers, and drinkers to carry out key monitoring and health intervention.

## Data Availability

The data supporting the findings of this study are not publicly available due to ethical and privacy considerations. Requests for data access can be made to the corresponding author upon obtaining ethical approval.
