# Correction: The impact of occupational hazards in coking plants on the incidence of hypertension—a longitudinal study

**DOI:** 10.3389/fpubh.2026.1823157

**Published:** 2026-03-16

**Authors:** Wei Zhang, Shengyu Fan, Yifan Li, Tiantian Chen, Xingyu Peng, Hongmei Gu, Shu Guo

**Affiliations:** 1 State Key Laboratory of Environmental Pollution Health Risk Assessment, South China Institute of Environmental Sciences, Ministry of Ecology and Environment, Guangzhou, China; 2 School of Public Health, Mudanjiang Medical University, Mudanjiang, China; 3 School of Public Health, China Medical University, Shenyang, China; 4 Loudi Center for Disease Control and Prevention, Loudi, China

**Keywords:** benzene and benzene series, coking plant worker, dust, hypertension, noise, occupational hazards

An incorrect **Funding** statement was provided. The correct funder is “Ministry of Science and Technology of the People's Republic of China.” the correct funding statement reads:

“The author(s) declared that financial support was received for this work and/or its publication. This study was supported by a grant from the Ministry of Science and Technology of the People's Republic of China through the National Key Research and Development Program (No. 2023YFC3905105).”

The original version of this article has been updated.

